# Spatial and Temporal Variations in Aquatic Organic Matter Composition in UK Surface Waters

**DOI:** 10.1021/acsestwater.4c01113

**Published:** 2025-04-29

**Authors:** Catherine S. Moody, Nicholle G. A. Bell, C. Logan Mackay, Ezra Kitson

**Affiliations:** † water@leeds, School of Geography, University of Leeds, Leeds LS2 9JT, U.K.; ‡ School of Chemistry, University of Edinburgh, Edinburgh EH9 3FJ, U.K.

**Keywords:** FT-ICR MS, dissolved organic matter, elemental analysis, drinking water treatment, carbon

## Abstract

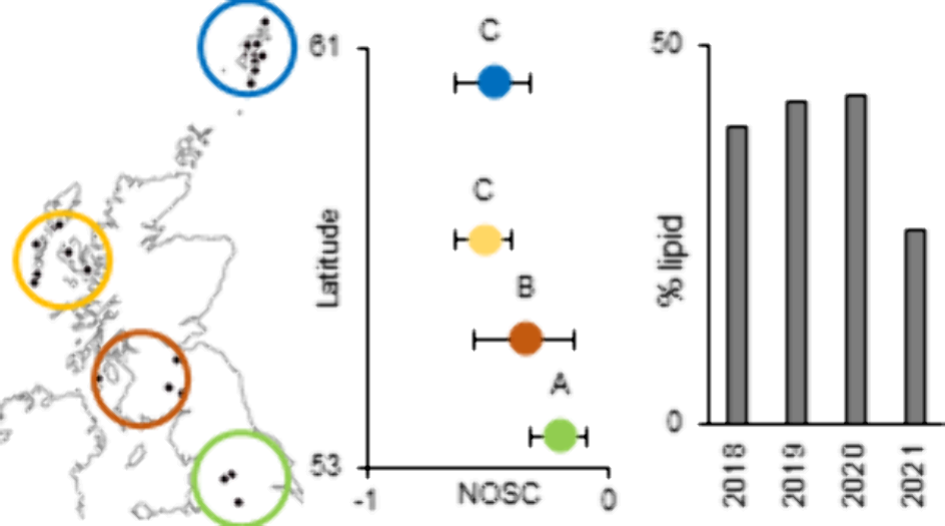

Drinking water is becoming more difficult to treat, especially in the UK, due to the changing concentration and composition of aquatic dissolved organic matter (DOM). The spatial and temporal variations in the DOM composition are not well understood. This study investigated how DOM composition varies along a north/south gradient in the UK, over four years, and between headwaters and reservoirs. There were trends in DOM composition metrics from north to south; carbohydrate and peptide-like compounds were lower in northern sites, while lipid-like compounds were lower further south, suggesting different sources of DOM in north/south catchments. DOM collected in Autumn 2021, after a Summer of low rainfall, was more aromatic, less oxidized, and more diverse than DOM collected in 2018–2020. Decreased lipid content and increased oxy-aromatic content occurred in Autumn, at the end of the plant growing season, when increased rainfall rewets catchments and mobilizes soil OM into surface waters. These seasonal changes in DOM composition coincide with increased DOM concentrations in raw drinking water, leading to more challenges for drinking water treatment, especially as climate change alters rainfall distribution in the UK.

## Abstract 2

Understanding natural variations in organic matter composition is important for UK drinking water treatment. This study used four years of data to identify trends in organic matter composition with latitude, climate, and time of year.

## Introduction

Decay of vegetation and peat leads to natural organic matter (OM) in waterways draining from peatland sites that are rich in carbon-containing compounds; OM from peat can contain between 44 and 70% carbon (C).^1,2^ Fluvial OM fluxes represent a significant C loss from peatland habitats to downstream systems and to the atmosphere.^3,4^

In temperate peatlands, OM inputs to aquatic systems vary seasonally due to climate-driven changes in vegetation growth and decay, affecting both concentration and composition of OM.^5^ OM concentrations in peatland waters are influenced by location, climate, weather, vegetation, and land use.^6−8^ Sea spray impacts peatland vegetation, decomposition, and soil OM, influencing terrestrial and aquatic OM near coastlines.^9,10^ The UK’s maritime climate is changing, with increasing summer temperatures and decreasing annual rainfall expected to alter peatland extent and OM concentrations.^4,11^ Additionally, rising sea levels around UK islands and more frequent storms will likely increase sea spray.^12^

Variable OM compositions lead to complex issues for drinking water suppliers. In the UK, up to 70% of drinking water is sourced from peatland and upland environments, and these incoming waters contain high OM and organic C concentrations.^13,14^ Any residual OM present after drinking water treatment (DWT) can form potentially carcinogenic disinfection byproducts (DBPs), and so water companies must minimize the residual OM concentration in their water.^15^ Water companies know to expect seasonal variations in OM concentration (e.g., high concentrations in Autumn, due to plant dieback after Summer growing season), but seasonal changes in OM composition, and their impact on DWT processes, are less well understood.^16^ Water entering DWT plants has become more difficult to treat, with water companies reporting that OM concentrations have risked exceeding the capacity of treatment works, especially in reservoirs on peat soils, and an increase in DBPs in coastal and island reservoirs.^17,18^ To continue providing clean and safe drinking water, water companies need to know more about OM composition in their raw water sources, how it varies over time in their supply area, and how to remove it effectively and efficiently.

Dissolved organic matter (DOM, fraction smaller than 0.45 μm) is comprised of thousands of different compounds of various origins.^19^ In peatland headwater streams, compounds are from biodegradation of vegetation, peat and photodegradation products, and from physical erosion of peat, and contain aromatic compounds, lipids, carbohydrates, peptides, amino acids, and sugars.^2,20^ The relative contribution of these different compounds impacts bioavailability and therefore is important in determining microbial productivity and reactivity^21^ and its treatability during DWT.^22^

Analytical methods have been used to understand more about aquatic DOM composition (e.g., UV–vis, fluorescence, elemental analysis, and nuclear magnetic resonance^23−25^). Techniques such as Fourier transform ion cyclotron resonance mass spectrometry (FT-ICR MS) give much more detail of the molecular composition of complex mixtures.^7,17,26−29^ Advances in compound libraries and analysis have made FT-ICR MS more accessible, making it possible to analyze and interpret data from more samples in a short amount of time (e.g., Kitson, Kew, Ding, and Bell^30^).

DOM composition metrics can be calculated from elemental content of carbon (C), hydrogen (H), nitrogen (N), and oxygen (O) and the molecular formula. C/N and oxidative ratios give indicators about DOM treatability and oxidation state, and H/C and DBE (double-bond equivalent) give indicators about DOM structure and reactivity.^15,16^ The molecular formula allows ‘molecular richness’ diversity metrics to be calculated^31^ and compounds classes to be assigned, including lipids, carbohydrates, peptides, amino sugars, oxy-aromatic phytochemicals, and nucleotides.^32^

The aim of this study was to determine how DOM composition from peatland surface waters varies over space and time in the UK. Water and DOM samples were collected either monthly or yearly from sites within drinking water catchments in four geographical areas and analyzed to find their composition using elemental analysis and FT-ICR MS. Specifically, we hypothesized the following:

1. DOM composition and molecular diversity would be different at geographic areas of the UK, related to mean annual temperatures, rainfall, and marine influence (e.g., island and mainland locations) impacting on DOM source materials.
2. DOM composition and molecular diversity would vary over time, with interannual and intra-annual trends, due to interannual variations in climate and intra-annual variations in vegetation cycles and seasonal weather.

## Materials and Methods

### Study Sites

**Figure 1:**
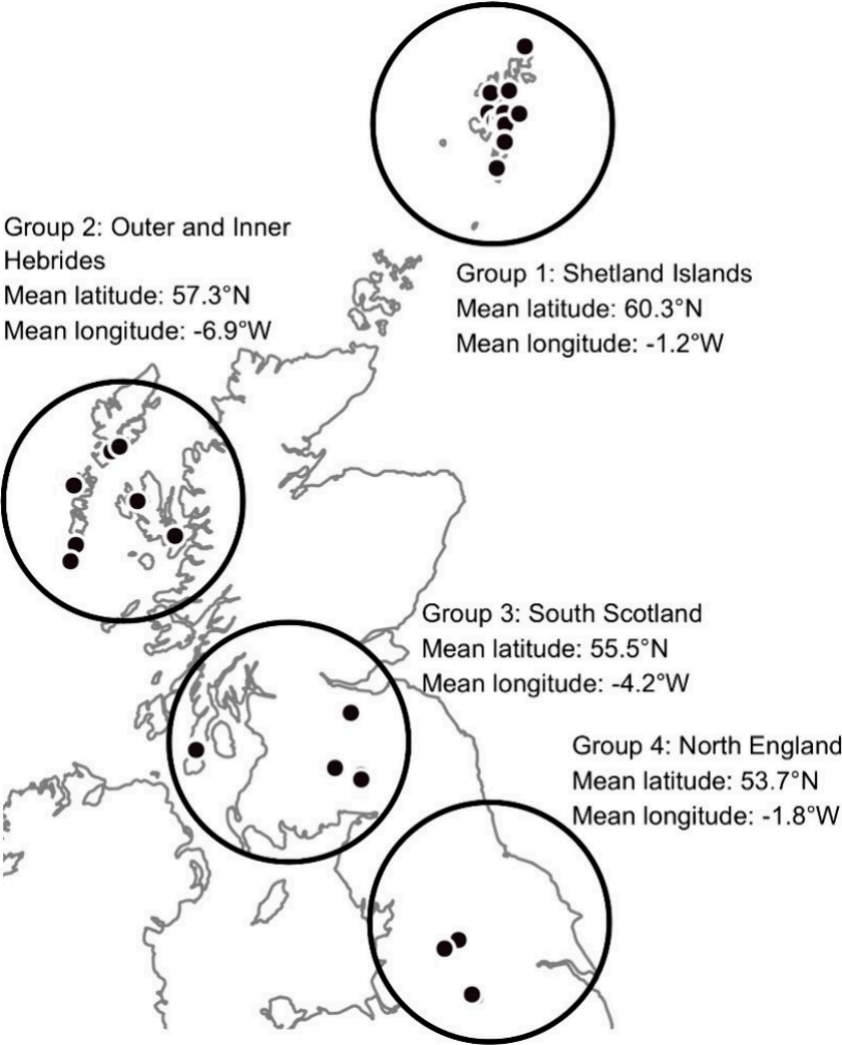
Locations of sites used in this study. Some symbols represent more than one site as they were too close to separate at this scale. The number of sites in each group is in Table 1.

There were 192 water samples, from 41 individual sites in upland areas across the UK. Sites were visited up to 24 times between 2018 and 2021, and water samples were collected and analyzed from 28 catchments (some catchments contained more than one site; Figure 1, Table 1). Water companies are keen to understand remote island drinking water supplies, as they are particularly vulnerable to changes in DOM concentration and composition, and so several sites on islands were included in this study. Locations were chosen in four distinct groups with different mean annual temperatures (MAT) and rainfall (MAR) to determine the impact of location, climate, and distance to the sea on DOM (Table 1).^33^ Drinking water supplies from catchments with peat soils on Shetland Islands (Group 1, highest latitude, coolest max MAT, close to the sea), the Inner and Outer Hebrides (Group 2, furthest west, close to the sea), Borders and Argyll and Bute (Group 3, highest MAR), and Yorkshire Dales and Peak District (Group 4, highest MAT, lowest MAR, lowest latitude) regions were included in the study. Water was collected from two or three sites within each catchment, in collaboration with water company partners, and within access constraints. Meta-data for each site, including catchment area, percentage peat cover, land use and vegetation cover, and distance from the sea, was assigned based on observations at the site or derived from publicly available databases and maps. See the Supporting Information for more detail.

**Table 1:** Groups, Number of Sites and Number of Samples in Each Group, Frequency of Visits, and Site Information^a^

**Variable**	**Longitude(°W)**	**Latitude(°N)**	**Elevation(m asl)**	**Catchment area(km**^**2**^**)**	**Peat(%)**	**Distance to the sea(km)**	**Mean annual air temp(°C)**	**Mean annual rainfall(mm**)
N	41	41	41	41	41	41		
Min.	–7.45	53.43	–0.76	0.03	52.3	0.10	5.53	
Max.	–0.90	60.82	401.00	47.13	100	70.5	12.79	
Mean	–3.30	57.88	131.94	5.88	90.3	11.23		1162.93
Std. Er.	0.38	0.38	19.30	1.54	1.5	3.04		
**Group 1, Shetland Islands*n* = 18, visited yearly, 55 DOM samples**								
Mean	–1.21	60.32	61.76	3.29	92.4	2.04	Min:5.60	1252.34
Std. Er.	0.04	0.04	12.57	1.02	2.7	0.35	Max:9.80	(Lerwick)
**Group 2, Outer and Inner Hebrides*n* = 10, visited yearly, 30 DOM samples**								
Mean	–6.90	57.33	62.78	1.42	89.6	1.78	Min:6.17	1235.52
Std. Er.	0.19	0.11	17.54	0.53	3.0	0.30	Max:11.50	(Stornoway)
**Group 3, S. Scotland (Borders, Argyll and Bute)*n* = 9, visited yearly, 36 DOM samples**								
Mean	–4.16	55.48	267.78	16.95	86.3	21.12	Min: 4.07	1827.17
Std. Er.	0.37	0.07	32.61	5.27	2.3	4.31	Max: 11.40	(Eskdalemuir)
**Group 4, N. England (N. Yorkshire, Peak District)*n* = 4, visited quarterly/monthly, 71 DOM samples**								
Mean	–1.79	53.66	315.05	3.78	91.8	68.27	Min:6.92	831.55
Std. Er.	0.10	0.13	42.42	2.83	4.6	1.40	Max:13.71	(Sheffield)

a Elevation (m asl) = meters above sea level, Peat (%) = proportion of the whole catchment covered in peat. Mean annual maximum and minimum air temperatures and total rainfall (30-year averages, 1991–2020) from the UK Met Office Climate Data Portal, from met stations in each area.

### Sampling Frequency

The sites were revisited between 2018 and 2021, yearly, quarterly (every 3 months), or monthly (Table 1) and were therefore subject to different weather and flow conditions. Underlying geology has been shown to impact groundwater contributions to peat streams and rivers at baseflow conditions;^34^ the annual samples were collected during Autumn when flows were high; therefore the impact of underlying geology on annual samples was considered minimal. Flow conditions varied during quarterly and monthly sampling at the Group 4 sites. Sampling was disrupted by the covid-19 pandemic lockdown in Spring/Summer 2020, during which only sites local to the authors could be sampled. Due to variable reservoir and lake surface water levels, the exact sampling location varied by up to 5 m (up/down slope).

### Sample Collection and Water Chemistry

Water pH, electrical conductivity, dissolved oxygen and temperature (Hach MM156 portable multiparameter meter), and air temperature, pressure, and humidity were recorded at the time of water sample collection. Two water samples were collected at each site. A small sample (50 mL) was immediately filtered (0.45 μm), stored in a cool box (in the dark), and later analyzed for DOC, total nitrogen, total phosphorus, dissolved nutrients, metal and ion concentrations, and absorbance. A large sample (approximately 5 L) was filtered (0.7 μm), particulate organic matter (POM) was collected and rotary evaporated, and DOM was collected (using the method from Moody^25^). For all analysis methods, a subset of samples was analyzed in replicate, and certified reference materials were used to calibrate equipment.

### DOM Analysis

Solid DOM samples (n = 192), extracted from filtered water using low temperature rotary evaporation (<60 °C), were analyzed by elemental analysis (EA) for their carbon, hydrogen (H), nitrogen (N), oxygen (O), and organic C (C_org_) content (Elementar Vario MICRO cube). 10% of samples were analyzed in replicate and reanalyzed if the root-mean-square error of the replicates was less than 95%.

A subset of DOM samples (n = 77) was analyzed by negative-mode electron spray ionization (ESI) Fourier-transform ion cyclotron resonance mass spectrometry (FT-ICR MS). Twenty-four monthly DOM samples from Group 4 reservoir and headwater paired sites, 32 from four catchments in Group 3, 15 from two catchments in Group 2, and six from Group 1 catchment were included in this analysis. Samples were chosen based on sampling frequency and site and enough material available for analysis, to give more information about annual and seasonal variations in DOM composition.

0.5 mL of each sample was added to 0.5 mL of LC-MS grade methanol and centrifuged at 10,000 rpm for 5 min. 200 μL of the supernatant was then drawn into an analytical syringe and injected directly into the ESI source. The FT-ICR MS analysis was conducted on a 12T Bruker Solarix at the University of Edinburgh, SIRCAMS facility. The following tuning parameters were used: flow rate 120 μL h^–1^, capillary voltage 4500 V, low mass cut off 100 m/z, high mass cut off 3000 m/z, ion accumulation time 0.2 s, and time-of-flight 0.7 ms. In each case, 200 scans at 8 MW were summed.

The FT-ICR MS output data were processed using the CoreMS Python library (Corilo, Kew, and McCue;^35^https://github.com/EMSL-Computing/CoreMS). Briefly, raw spectra were peak-picked in the range 100 to 700 m/z after applying a noise-threshold based on the log-intensity distribution of each spectrum^36^ and a minimum peak prominence filter of 0.01%. Next, internal calibration was performed with a second-degree polynomial fit against a reference peak list of CHO containing formula with double bond equivalent (DBE) of −1, 0, and 1, shown to be highly abundant in DOM.^28,37^ The ppm error thresholds for peak matching during internal calibration were predetermined by performing an unconstrained assignment of a CHO containing formula to each spectra and visualizing the intrinsic error distribution (i.e., the relationship between *m*/*z* error and *m*/*z*). Following internal calibration, a constrained formula assignment was performed using the following elemental constraints: C 1–90, H 4–200, O 1–26, N 0–2, and S 0–1 and an *m*/*z* error tolerance of ±0.5 ppm. Phosphorus was not included in elemental constraints as the ionization efficiency of P molecules during FT-ICR MS is very limited, and adding P to formula assignments increases the number of false assignments. Finally, formulas detected in blank methanol samples with a prominence of more than 20% were removed from samples acquired on the same day as the blank. Assigned peak lists were then imported into the PyKrev Python library for analysis.^30^

### Data and Statistical Analysis

CHNO and organic C molar concentrations (from elemental analysis and FT-ICR MS) were used to calculate derived metrics: %C_org_ (organic C portion of total C), DBE/C (double bond equivalent per carbon, SI eq 1), C_ox_ (carbon oxidation state, SI eq 2), OR (oxidative ratio, SI eq 3), NOSC (nominal oxidation state of carbon, SI eq 4), C/N, H/C, O/C, and AI (aromaticity index, SI eq 5).^27,38,39^ NOSC, C_ox_, and OR indicate oxidation state, with negative NOSC and C_ox_ values representing reduced compounds.^40^ DBE/C and AI reflect aromaticity, while %C_org_ estimates organic soil contribution. Molar ratios help classify compounds and determine degradation state.^24^ FT-ICR MS data were also used to assign molecules to compound classes (lipids, carbohydrates, peptides, amino sugars, oxy-aromatic phytochemicals, and nucleotides) based on stoichiometry^32^ and calculate molecular richness.^31^ See the Supporting Information for details.

DOM composition metrics were analyzed in a general linear model (GLM). Samples collected in Autumn (Sep, Oct, and Nov), at all sites and groups and across all four years, were included (Table S1). Results are reported as significant if the p value is less than 0.05. Posthoc Waller-Duncan k-Ratio *t* tests showed differences within significant groups and years. Linear regressions were used to find relationships between DOM composition metrics and possible explanatory factors, including air and water temperatures, distance to sea, and latitude. Results are reported as adjusted R^2^ (adj. R^2^) values.

The seasonal cycle of DOM composition metrics was investigated using Group 4 DOM samples, in each calendar month (January = 1, Dec = 12) and in each UK season (Winter = Dec, Jan, Feb; Spring = Mar, Apr, May; Summer = Jun, Jul, Aug; Autumn = Sep, Oct, Nov), using a repeated measures GLM. To investigate seasonal and within-catchment differences in diversity, the molecular formula data for two Group 4 sites (a headwater stream and reservoir surface water within the same catchment, visited monthly for a year) were compared. Compounds unique to either site (headwater or reservoir) or sampling month (Jan-Dec) were identified. This provides an estimate of diversity, by identifying how ‘unique’ each DOM sample is.

## Results and Discussion

### Spatial Analysis

**Figure 2:**
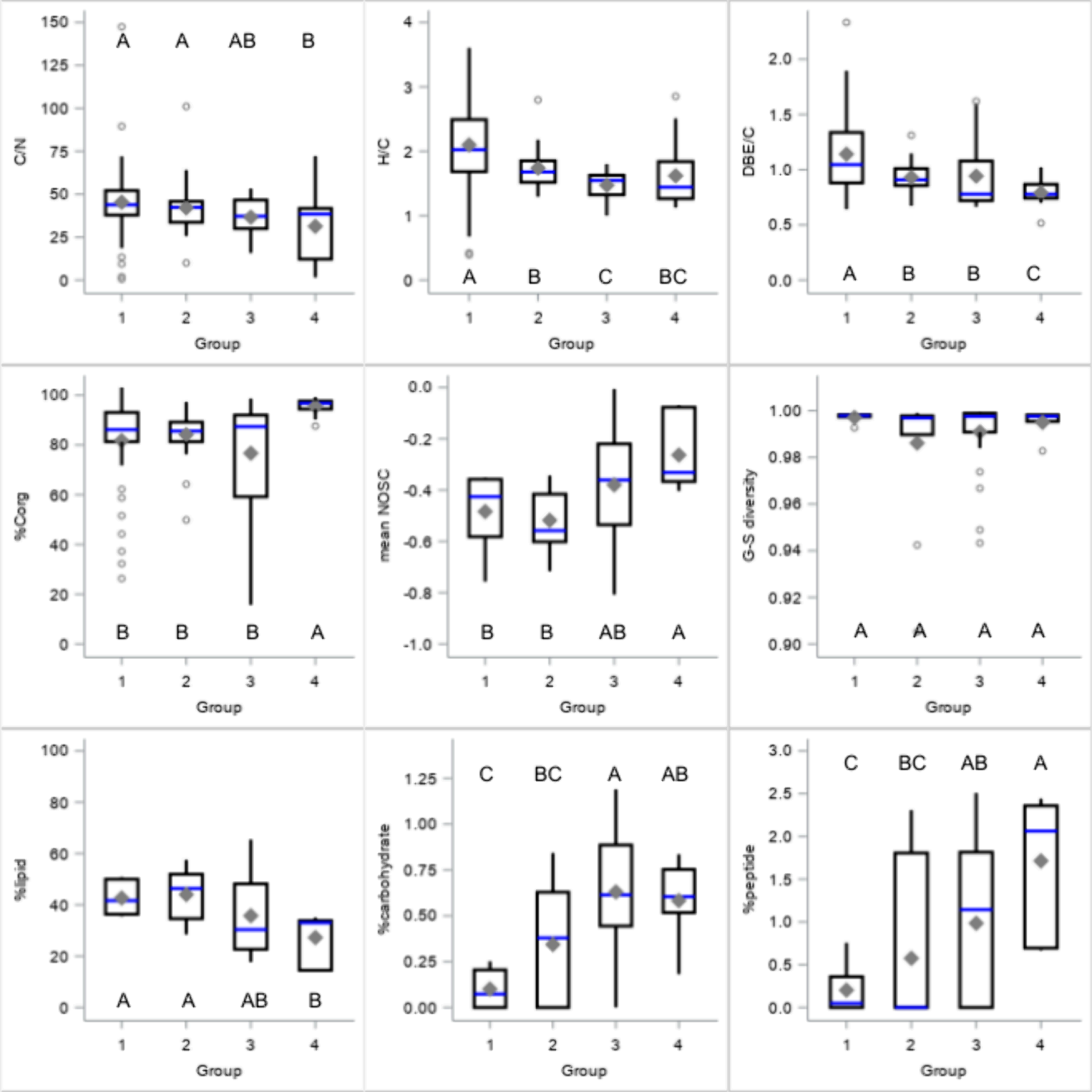
Box plots of DOM composition metrics for groups 1–4. Groups: 1 = Shetland, 2 = Hebrides, 3 = South Scotland, and 4 = North England. Letters indicate significant differences.

Most DOM composition metrics differed significantly between groups, but group differences only explained 1–28% of the variation (GLM, partial R^2^). No metric was significantly different across all four groups, although there were some spatial trends. Group 1 DOM had higher H/C and DBE/C but lower carbohydrate content (Figure 2). Groups 2 and 3 DOM had intermediate values, differing significantly in H/C and carbohydrate content. Group 4 DOM had higher %C_org_ and NOSC but lower lipid content, C/N, and DBE/C. Overall, DOM from island sites (Groups 1 and 2) had lower carbohydrates and NOSC but higher lipid content than mainland sites (Groups 3 and 4).

Latitude and air or water temperature explained 4–23% of variation in DOM composition and molecular diversity (linear regression, *p* < 0.05). Gini-Simpson diversity decreased with increasing air temperature (adj. R^2^ = 13%, p = 0.0114), but Shannon–Wiener values showed no significant relationship with temperature or latitude. C/N, oxygen state (O/C, C_ox_, OR), and peptide content were significantly related to the distance from the sea (lake and reservoir DOM, linear regression, adj. R^2^ = 11–49%). However, across all annual samples, distance to the sea explained at most 9% of variation and showed no significant relationship with DOC concentration. Models including six physical location parameters explained up to 29% of DOM composition variation (Table S2).

Spatial variation in DOM composition differed significantly between northern and southern UK sites, supporting the hypothesis of spatial differences. However, these were not solely explained by location (island vs mainland) or strongly linked to latitude, distance to sea, or temperature, despite their relevance to DOM decomposition and plant growth.^9,41^ Northern sites had lower carbohydrate, oxy-aromatic, and peptide compounds, while lipid content was lower further south, indicating different DOM sources. Low-lipid samples likely originate from terrestrial (plant and peat) sources, whereas high-lipid samples suggest microbial origins.^42^ DOM in northern sites was more oxidized and had fewer double bonds per C (more saturated) than further south, suggesting it would degrade more readily at northern sites (Moody and Worrall 2017; Leifield et al. 2020).

Vegetation cover differed between island and mainland sites with no trees on the islands. As a key source of terrestrial DOM, vegetation influences DOM composition.^5^ Removing vascular plants from peatlands increased humic to lignin ratios and decreased aliphatic to polysaccharide ratios, due to changes in root exudates,^43^ and planting trees reduced soil organic C in moorland soils.^44^ However, in this study, vegetation cover explained only 14–17% of the variation in a few DOM composition metrics (Table S2). Site land, which correlated with vegetation cover, was significant for C/N, H/C, and AI, explaining 15–23% of variation (Table S2).

Soil is a major source of aquatic DOM, and all of the study sites had at least 50% peat cover. Group 4 sites (low lipid content) were likely dominated by terrestrial inputs, with 81–100% peat cover. Some Group 1 sites had nearly 50% freshwater cover, where microbial activity could increase DOM lipid content.^20,42^ However, the lipid content was not significantly related to peat cover, indicating a more complex DOM-peat relationship.

Studies by Roth, Dittmar, Gaupp, and Gleixner,^41^ Zhu, Zhao, Bai, Zhou, Chen, and Wei,^45^ and Verbeke, Lamit, Lilleskov, Hodgkins, Basiliko, Kane, Andersen, Artz, Benavides, and Benscoter^46^ found significant DOM differences across latitudes (79°N–65°S) and attributed these to temperature, vegetation cover, and DOM degradability, with implications for drinking water treatment. The differences in DOM composition across latitude, vegetation cover, and land use show the challenge facing water companies, especially those with large catchments, to treat incoming raw water with highly variable DOM compositions and concentrations. However, the differences in DOM compositions in this study explained by Group, latitude, distance to sea, and temperature were at most 28%, highlighting the need to consider other factors, such as the impact of riparian zone peat and vegetation cover. For example, in large reservoir catchments, the vegetation cover of a distant part of the catchment may have minimal impact on the in-reservoir DOM composition compared to vegetation in the reservoir riparian zone. Small streams may have 100% peat in their riparian zone but have lower % peat cover across the whole catchment, leading to lower explanatory power of peat cover in models.

### Temporal Analysis - Annual

**Figure 3:**
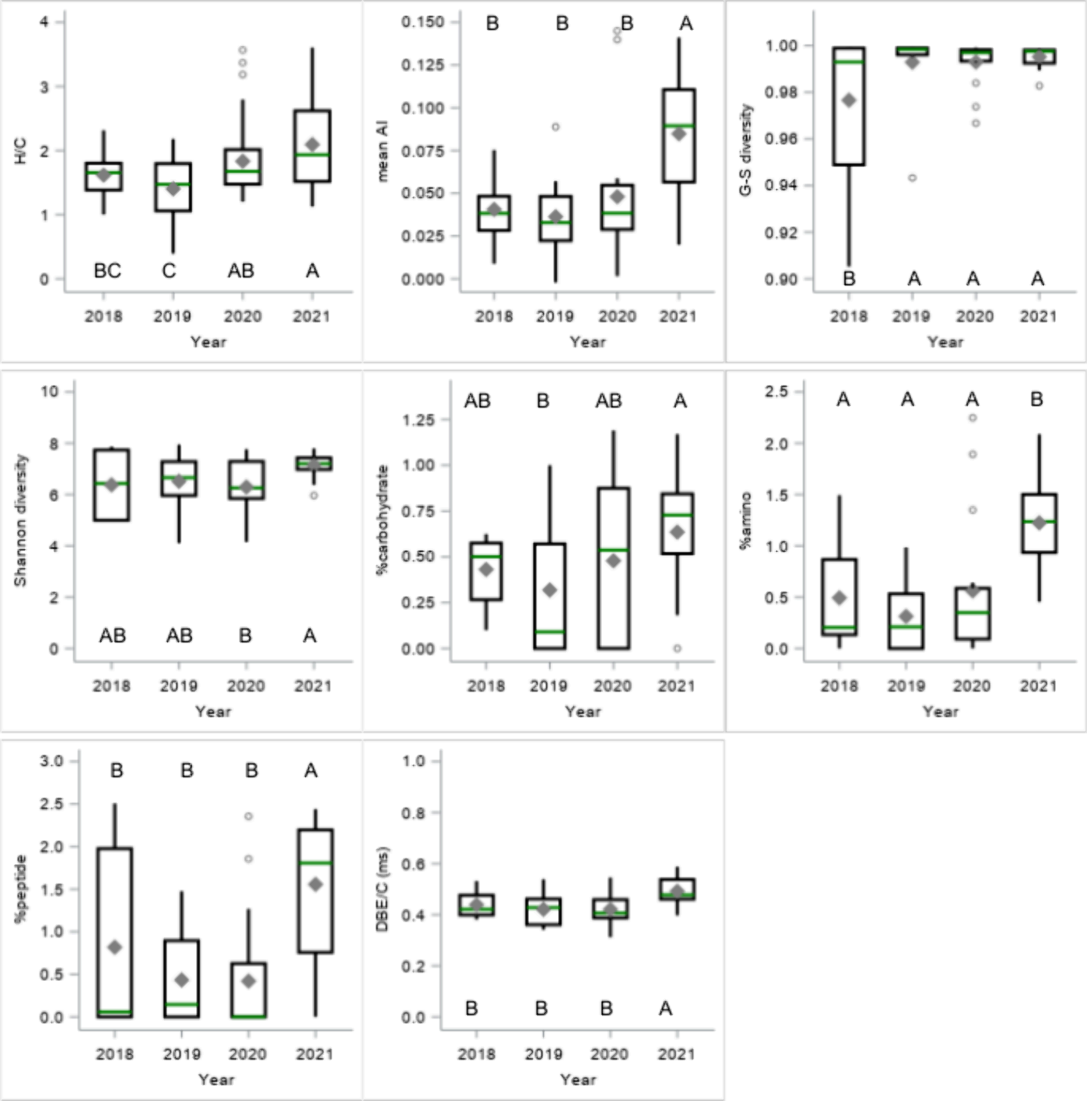
Boxplots of DOM composition metrics for years 2018–2021. Letters indicate significant differences.

Annual comparisons of Autumn DOM samples showed significant interannual differences in most composition metrics (GLM, partial R^2^ 1–44%, Table S1), though no metric differed across all four years. Shannon–Wiener diversity, oxy-aromatic content, and C/N ratios remained stable. Notably, 2021 samples were more aromatic, less oxidized, and more diverse, with high amino sugars and peptide content but lower lipid content than 2018–2020 samples (Figure 3).

Shatilla and Carey^47^ linked interannual DOM variation to rainfall and temperature, where high rainfall led to high stream discharge and high DOC concentrations, but inversely affected SUVA and fluorescence indices. Verbeke, Lamit, Lilleskov, Hodgkins, Basiliko, Kane, Andersen, Artz, Benavides and Benscoter^46^ found MAT influenced carbohydrate and aromatic content in DOM, alongside latitude and altitude.

Despite interannual variations in UK temperature and rainfall,^48,49^ 2021’s annual values were not extreme (Figures S1A, S1B). Scotland’s Autumn (Sep-Nov) rainfall quantities were consistent across 2018–2021 (382–527 mm; Figure S1C). However, Summer rainfall varied widely, from 197 mm in 2021 to 436 mm in 2019 (Figure S1C), with 2021 also experiencing the driest September (Figure S1D). Drought alters plant root exudates, influencing soil C cycling and decomposition.^8,14,50^ In UK peatlands, late-Summer rainfall mobilizes soil OM into surface waters;^5^ therefore dry summers, such as the Summer of 2021, may yield distinctly different aquatic DOM.

### Temporal Analysis - Seasonal

**Figure 4:**
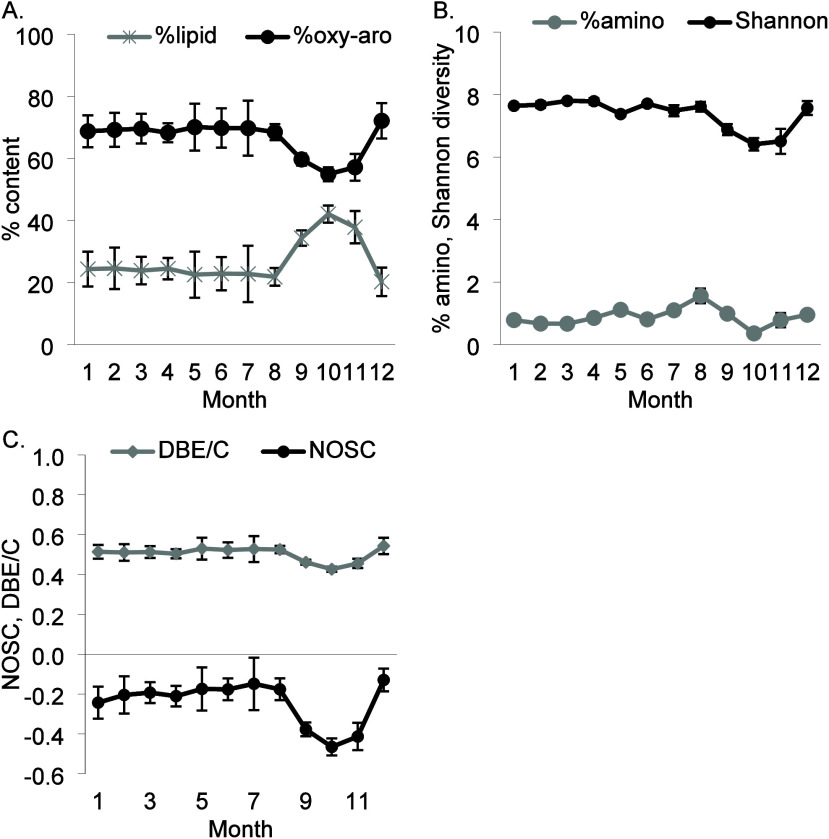
Mean and standard errors of DOM composition metrics for months 1–12 (1 = Jan, 12 = Dec). A) lipid (GLM repeated measures *p* = 0.01) and oxy-aromatic content (*p* = 0.03), B) Shannon–Wiener diversity (*p* = 0.03) and amino content (*p* = 0.01), C) DBE/C (*p* = 0.01) and NOSC (*p* = 0.01).

Monthly analysis of Group 4 DOM samples showed significant seasonal shifts in composition (DBE/C, NOSC, Shannon–Wiener diversity, %lipid, %amino, and %oxy-aromatic content). Most changes occurred in late Summer and Autumn (Figure 4, Figure S2). Oxy-aromatic content remained stable (∼70%) from January to August and then decreased to 55% in Autumn, before recovering in December. Lipid content showed an inverse trend, rising from ∼20–25% (January to August) to 34–42% in Autumn. AI varied but stayed low during Autumn. The C/N ratio increased from 25 in Spring to 41 in Autumn before slightly decreasing in Winter. C_ox_ peaked in Spring (April = 1.97) and Summer (August = 1.48), while OR showed an inverse pattern. Amino sugar content followed a trend similar to that of C_ox_, peaking in May and August. Molecular diversity, NOSC, and DBE/C also decreased in Autumn.

The Shannon–Wiener diversity reduction coincides with an increase in lipid-like compounds, suggesting a shift in DOM composition. Autumn increased rainfall flushes freshly leached plant-derived DOM from plant senescence and decomposition.^51^ The H/C and O/C values indicate a greater contribution of lignin-derived compounds, while the reduced NOSC and decline in oxy-aromatic compounds support the interpretation that the DOM is less degraded and freshly mobilized from the soil. Additionally, toward the end of the growing season, microbial activity in the peat declines, leading to reduced degradation and oxidation of DOM.^52^

Wilske, Herzsprung, Lechtenfeld, Kamjunke, Einax, and von Tümpling^53^ showed monthly changes in molecular formula intensities in reservoirs in Germany. They discovered significant changes in DOM composition, such as aliphatic compounds with low molecular weights that were more intensive when the reservoir was stratified and were generally found at the surface, and during Summer, aromatic compounds with high molecular weight and high O content decreased. These changes impacted DWT, as highly unsaturated and O-rich compounds can be removed by coagulation with Fe or Al, whereas smaller products of photodegradation were precursors of DBPs. Wilske, Herzsprung, Lechtenfeld, Kamjunke, Einax, and von Tümpling^53^ and Chen, Uzun, Tolić, Chu, Karanfil, and Chow^40^ show that proxies for H/C ratios and DOM molecular weight could be useful for water companies to determine DOM removal efficiency, particularly via coagulation treatment.

### Spatial and Temporal Analysis within a Catchment

**Figure 5:**
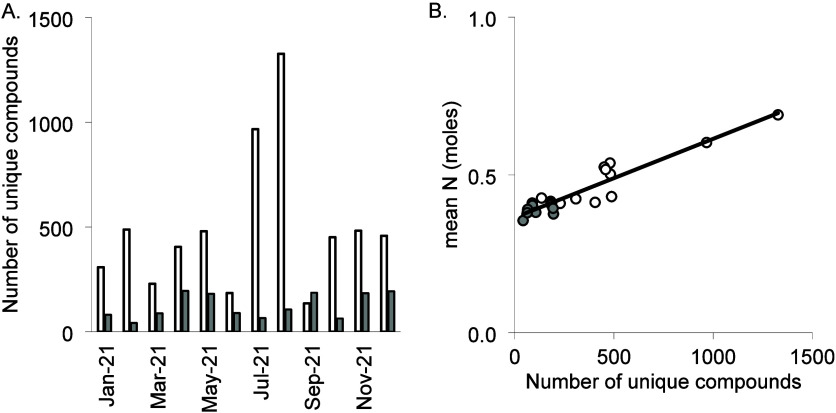
Number of unique compounds from the headwater (white) and reservoir (gray) A) on each sampling occasion with B) mean N content of DOM.

Headwater DOM had more individual compounds (average = 6,554) than reservoir DOM (average = 5,453; Figure S2) in the same catchment, suggesting higher DOM composition diversity in the headwater. Across both Group 4 sites, 7,388 compounds appeared only once in a single sample (Figure 5). Headwater DOM had more unique compounds (average = 493, 8% of total) than the reservoir (average = 123, 2.4% of total). Unique compounds in headwater DOM peaked in Summer, while reservoir DOM had the most in Spring. The high number of unique compounds in headwater DOM coincided with warmer temperatures and lower rainfall, though relationships were not significant. Increased Summer headwater DOM diversity could indicate a higher input of groundwater during baseflow conditions.^34^

There were several significant relationships between the number of unique compounds on each sampling occasion and other metrics (DOM composition and water chemistry), explaining 16–88% of the variation (Table 2). DOM samples with high numbers of unique compounds had low lipid, peptide, and H content, low H/C, O/C, and C_ox_ values, and low pH and cation (Ca, K, Mg, and Na) concentrations. This indicates that the increase in unique compounds during Summer (e.g., baseflow conditions) is not due to groundwater input, as groundwater has higher cation concentration (specifically Ca, Na, and Mg), electrical conductivity, and higher pH than peat streamwater.^34^

**Table 2:** Relationship between the Number of Unique Compounds in All 24 Samples with DOM Metrics and Water Chemistry Metrics^a^

	**Variable(y)**	**n**	**adj R**^**2**^	**p**	**Intercept**	**Count of unique compounds parameter estimate(x)**
EA	C/N	24	0.43	0.0003	14.38	0.03527
EA	C_ox_	23	0.20	0.0174	1.78	–0.00121
EA	H/C	22	0.33	0.0029	2.50	–0.00146
EA	O/C	15	0.43	0.0050	1.57	–0.00079
EA	OR	23	0.16	0.0355	0.61	0.00025
MS	%amino	24	0.49	0.0001	0.68	0.00097
MS	%carb	24	0.22	0.0116	0.50	0.00031
MS	%lipid	24	0.52	0.0001	30.10	–0.01899
MS	%oxy-aro.	24	0.46	0.0002	63.00	0.01758
MS	%peptide	24	0.46	0.0002	1.93	–0.00142
MS	Mean AI	24	0.34	0.0016	0.06	0.00008
MS	Mean C	24	0.60	0.0001	21.28	0.00334
MS	Mean H	24	0.38	0.0008	24.68	–0.00253
MS	Mean *m*/*z*	24	0.61	0.0001	411.46	0.06774
MS	Mean N	24	0.88	0.0001	0.37	0.00025
MS	Mean NOSC	24	0.56	0.0001	–0.30	0.00030
MS	Mean O	24	0.37	0.0009	7.56	0.00132
MS	DBE/C	24	0.53	0.0001	0.48	0.00013
Water	%DON	22	0.39	0.0010	32.73	0.05446
Water	DOC	24	0.63	0.0001	4.80	0.04634
Water	DON	22	0.61	0.0001	0.18	0.00092
Water	Alkali and AEM	24	0.31	0.0027	17.95	–0.01502
Water	Heavy metals	24	0.42	0.0004	0.44	0.00153
Water	pH	20	0.48	0.0004	6.45	–0.00294

a Alkali and alkaline earth metals (AEM) = Ca, K, Mg, and Na; Heavy metals = Al, Fe, Pb, Co, Cu, and Zn.

DOM samples with high numbers of unique compounds had a high DOC concentration; this shows that water with high DOC concentrations is more likely to have more unique compounds. These DOM samples also had high C, O, N (Figure 5B), carbohydrate, amino sugar, and oxy-aromatic content and high C/N and OR. Water with high heavy metal and DON concentrations and a high proportion of organic N (%DON) also had DOM with a high number of unique compounds. These higher values also coincide with the end of the growing season and a change in weather (the Summer of 2021 had lower than average rainfall in the UK^49^), when compounds that have built up in the soil over a dry Summer will be mobilized into the water as the catchments rewet.^54^

Using the molecular composition data showed the number and complexity of DOM compounds (similar to Liu, Tan, Fang, Chen, Tang, Liu, and Yu^55^ and Cooper, Chanton, D’Andrilli, Hodgkins, Podgorski, Stenson, Tfaily, and Wilson^29^) found in reservoir water that are not present in headwaters, to show how much in-stream processing occurs in streams. The results also show that the number of unique compounds in a water can be directly related to water chemistry variables, with important implications for carbon cycling and DWT. Water chemistry and DOM metrics can give indications about how many unique compounds are likely to be in a water body, which can be used as a proxy for turnover of DOM – a water body with a high number of unique compounds with high *m*/*z* will have high DOC concentration and therefore lots of DOM available for photo and bio degradation and will therefore have a high turnover. Water with a low number of unique compounds has a more ‘stable’ and less varied composition, with low *m*/*z* and low DOC, and therefore is likely to be more refractory and have a lower DOM turnover.^39,55^

### Implications for Drinking Water Treatment

Water companies need to know the DOM composition in their incoming water supplies, so combining these results with findings from other studies (e.g., Moody^56^) will allow water companies to predict their composition envelopes and build treatment plans to manage with the variations likely to occur. This study showed significant changes in molecular-level DOM composition over time and space. However, smaller molecules (below the low mass cut off for FT-ICR MS 100m/z) such as short-chain organic acids and amino acids, small peptides, sugars, and phenol from plant decomposition and microbial metabolism were not included in this analysis.^57^ Including metrics derived from elemental analysis ensured these compounds were included in measures of DBE/C, Cox, etc. and show the benefit of analyzing DOM by more than one method.

In the UK, water companies use water color and SUVA_254_ to determine the best methods for treating drinking water ‘envelopes’. SUVA_254_ is a good predictor of precursors of DBPs in water treatment.^58^ DBP-precursors with high SUVA_254_, C/N, and C/O values resulted from C-rich DOM with high molecular weight and aromatic structures. DOM that was rich in N or O and lower in C resulted in DBP-precursors with low SUVA_254_ values. Only extremely low SUVA_254_ values resulted in low yields of DBPs. Hua, Chao, Huang, and Huang^58^ conclude that SUVA_254_ is a useful parameter for water companies, but it should be used in combination with other indicators, especially when SUVA_254_ is low, as it is not necessarily colinear with DOM composition or DOC concentration in water from varied locations, as it overlooks UV-inactive DOM components.^59^ Combining SUVA_254_ measurements with high resolution techniques such as NMR or FT-ICR MS will allow water companies to understand and treat their incoming DOM more efficiently.

The seasonal fluctuations in DOM composition found in reservoirs (such as changes in DBE/C, molecular diversity, and C/N) in this study would likely result in changes to the DBP-precursors and could lead to issues for DWT capabilities, especially when further enhanced by increased DOC concentrations during late Summer/early Autumn. DOM seasonal changes (and their impact on DWT) have been reported in countries with wet/dry seasons (e.g., Australia^60^ and Bangladesh^61^) and snowmelt (e.g., USA).^62^ Autumn leaf fall was a significant component in changing DOM composition in forested catchments in Maryland, USA.^63^

Shi, Zhuang, Hur, and Yang^15^ show how each DOM metric can give information about potential DWT efficiency e.g. compounds with high DBE and NOSC are adsorbed by ferrihydrite, whereas DOM with more lipid-like compounds were degraded by RuO_2_/Ti electrolysis. Smith, Moore, Semiao, and Uhrín^64^ used FT-ICR MS to show that ceramic membrane filtration significantly decreased aromatic and highly oxygenated DOM compounds (most likely to form DBPs), using unique compound analysis to determine the differences between raw and treated water. These studies demonstrate that with enough information about the DOM composition treatment processes can be targeted at specific types of DOM, resulting in lower DBPs and more efficient drinking water treatment.

## Conclusions

The results of this study show DOM composition varied spatially across 8° latitude, between the north of Scotland and mid-England in the UK, and temporally, between 2018 and 2021, both inter- and intra-annually. These differences were likely related to differences in Summer and Autumn rainfall trends and plant senescence at the end of the growing season. During 2021, when there was lower Summer rainfall, DOM was more aromatic, less saturated, and more diverse; these compounds could be absorbed by hematite nanocrystal adsorbent or coagulation during drinking water treatment (Shi et al. 2021). Higher rainfall (e.g., 2019 and 2020) resulted in DOM with lower diversity and peptide content, low H/C and higher lipid content, and lower NOSC. These samples were more reduced and would be removed via sand filtration. This study also showed that no simple model could explain or predict fluctuations of spatial and temporal DOM, highlighting the need to further investigate the drivers of the identified differences. As water companies are finding it more difficult to supply consistently high quality and quantity of water as the climate changes, these results can help determine future trends in DOM composition and steer water treatment priorities and requirements.

## Data Availability

Data are available from the NERC EIDC at https://doi.org/10.5285/ada28810-040b-4fef-8669-b21bac64a10b.

## Supporting Information Available

Supplementary methods (study sites, sampling frequency, sample collection and water chemistry, and DOM composition metrics) and results (spatial analysis and temporal analysis) (PDF)
