# Supplementary material for: Spatial and Temporal Variations in Aquatic Organic Matter Composition in UK Surface Waters
Source: ACS ES T Water. 2025 Apr 29;5(5):2233–43. doi: 10.1021/acsestwater.4c01113 (PMC12070417; doi:10.1021/acsestwater.4c01113)
Supplement: Supplementary file 1 — ew4c01113_si_001.pdf [file ew4c01113_si_001.pdf]

Supplementary Information: Spatial and temporal variations in aquatic organic matter composition in UK surface waters

Catherine S. Moody[a*], Nicholle G. A. Bell[b], C. Logan Mackay[b,c], Ezra Kitson[b,d]
[a] water@leeds, School of Geography, University of Leeds, Leeds, LS2 9JT, UK
[b] School of Chemistry, University of Edinburgh, Edinburgh, EH9 3FJ, UK
[c] Present address: Rosalind Franklin Institute, Didcot, Oxfordshire, OX11 0QX, UK
[d] Present address: UK Centre for Ecology & Hydrology, Bush Estate, Penicuik, Midlothian, EH26 0QB, UK
* Email: c.s.moody@leeds.ac.uk

Methods

Study Sites

Location: Latitude, longitude and elevation were recorded at each site using GPS. There were 37 sites in Scotland (28 sites on islands and 9 sites on mainland), and four in England. Sample collection ranged from 53 to 61°N, and -7 to -1°W, and from 0 to 400m above sea level.

Group 1 sites (n=18) were on the Shetland islands, and were generally low altitude lake inlets, lake surface waters, pools, reservoir inlets, reservoir surface waters and streams with small catchment areas (max 14km^2). There were no sites with trees, therefore no forestry land uses. The moorland at these sites was used for animal grazing and/or peat cutting, and small areas had been restored (gullies have been blocked to slow flow of water and re-wet sites). Vegetation cover was mainly blanket bog, and a few sites had heather or improved grassland cover. These sites were sampled annually, at least twice and at most, four times. The distance to the sea varied from 0.65 to 5.70km.

Group 2 sites (n=10) were on the Inner and Outer Hebrides islands, and were generally low altitude lakes (inlets, surface waters and outlets), reservoirs (surface waters and outlets), peat pools and streams with small catchment areas (max 5km^2). Land uses included peat cutting and moorland grazing (by sheep and deer). Vegetation cover was predominantly heather grassland, improved grasslands and blanket bog. These sites were sampled annually, between three and four times. The distance to the sea varied from 0.10 to 3.20km.

Group 3 sites (n=9) were in the south of Scotland and were all within four large drinking water reservoir catchments (max 47km^2). Water samples were collected from headwaters, inlets, reservoir surface waters and outlets. There were no pools, lakes or streams in this group. Land uses included plantation and felled forestry, and moorland grazing. Vegetation cover was acid grassland, coniferous woodland, heather and heather grassland. These sites were sampled annually, between three and four times. The distance to the sea varied from 4.95 to 36.80km.

Group 4 sites (n=4) were in the north of England and were higher in altitude than the island sites, but with similar catchment areas (max 12km^2). This group included sites sampled most frequently, up to monthly. Land uses were moorland managed for shooting and grazing, and woodland. Vegetation covers were coniferous woodland, heather and heather grassland. The distance to the sea varied from 65.70 to 70.50km. There were two sites – a headwater and its corresponding reservoir – that were visited seasonally (10 times) then visited monthly (12 times), between July 2018 and December 2021 (22 times in total). A small moorland stream in another catchment was visited monthly between November 2019 and December 2021 (24 times in total). There were breaks in sampling during Spring and Summer 2020 due to covid-19 lockdown restrictions. There were 67 DOM samples (one sample was lost in transit), of which 24 were analysed further by FT-ICR MS.

Island areas (Inner and Outer Hebrides, Shetland) generally had smaller catchment areas, lower elevation and higher percentage peat cover than mainland locations. Areas with higher human populations (e.g. South Scotland: Argyll and Bute, Borders; Peak District) had reservoirs with larger catchment areas and more variable percentage peat cover.

Catchment area: calculated using digital elevation models and QGIS software, or from the UK Lakes Portal (for lake and reservoir surface water sites). Catchment area varied from 0.03 to 47km^2, however the majority of catchment areas (n=34) were smaller than 10km^2. There were only six catchments between 10 and 30km^2, and one with a large area of 47km^2.

Catchment percentage peat cover: calculated using catchment area and UK CEH hydrology of soil type (HOST) classifications. Values are the proportion of catchment area within each HOST cell (1km^2), and the sum of the 'peat' categories in that area. Percentage peat cover ranged from 52 to 100%.
Areas with high percentage peat cover and island sites were identified as priority areas for water company interests. Out of a total of 41 sites, 24 had 90-100% peat cover, and 39 had 70-100% peat cover. Two sites had less than 70% peat cover; both these sites had small catchment areas, and the remainder of the catchment was covered in water.

Types of water body: water was collected from up to three locations in each catchment:
- artificial pools: formed as a result of human activity, such as building roads or blocking peat drains, n=3
- streams: sites where water did not directly contribute to a sampled reservoir/lake, n=8
- lake inlets (n=5), surface waters (n=7) and outlets (n=2): inlets and outlets from within 1km of a lake (not artificially impounded). No lake headwaters were included in the study
- reservoir headwaters (n=2), inlets (n=4), surface waters (n=8) and outlets (n=2): headwaters more than 1km upstream of a reservoir, inlets and outlets from within 1km of a reservoir (artificially impounded).

Land use: the primary land use at each site was inferred from site observations, photographs and map features, and broadly fell into two categories: woodland and moorland.
- Three sub-categories within woodland: felled forest (sites where trees have been felled for forestry), plantation (sites planted with conifer trees for harvesting), woodland (sites with trees and no clear other land use)
- Five sub-categories within moorland: cutting (sites where peat has been harvested for fuel or compost, either commercially or for personal use), grazing (sites used for animal grazing, including sheep, deer and cattle), restored (sites where peatland restoration have been applied), shooting (sites used for game shooting, including grouse and deer), moorland (sites with no trees and no clear other land use)

Vegetation cover: derived from UK CEH land cover maps. Vegetation cover included acid grassland, blanket bog, broadleaf woodland, coniferous woodland, heather, heather grassland, improved grassland

Sampling Frequency

There were six sites visited only twice, resulting in 12 DOM samples. These six sites (in four catchments located in the Shetland Islands) were visited in autumn in 2020 and 2021. There were

32 sites visited three/four times, annually, resulting in 113 DOM samples, of which 53 were analysed further by FT-ICR MS. These 32 sites (in 22 catchments located across England and Scotland) were visited every autumn between 2018 and 2021. There were three sites (a headwater and its corresponding reservoir, and a stream) visited more frequently. The headwater and reservoir were first visited seasonally (10 times) before being visited monthly (12 times), between July 2018 and December 2021 (22 times in total). The third site, a small moorland stream in another catchment, was visited monthly between November 2019 and December 2021 (24 times in total). There were 67 DOM samples (one sample was lost in transit), of which 24 were analysed further by FT-ICR MS.

Sample collection and water chemistry

A 50mL water sample was collected and filtered (0.45μm Sartorius cellulose nitrate membrane filter, pre-washed in deionised water) on site, then stored a cool box (in the dark). This water was used to analyse:

Total dissolved carbon and DOC: using Analytik Jena Multi NC2100 combustion analyser

Water colour: absorbance at 665, 470, 465, 436, 400, 360, 265, and 254nm was determined using a Jasco V-630 UV/Vis spectrophotometer

Anions: Chloride, nitrate and sulphate were measured using Dionex ICS3000 Ion Chromatograph

Nutrients: dissolved ammonium-N, nitrite-N, nitrate-N and phosphate, and total nitrogen and total phosphorus (TN and TP, after alkaline persulphate digestion) were determined on a Skalar Sans++ Continuous Flow Auto-analyser

Cations and metals: the concentration of dissolved cations and metals (aluminium, cadmium, calcium, cobalt, copper, iron, lead, lithium, magnesium, manganese, nickel, potassium, sodium and zinc) were analysed using Thermo iCAP 7600 Duo: Inductively Coupled Plasma – Optical Emission Spectrophotometer (ICP-OES).

Water chemistry derived metrics: the water colour and DOC concentration data were used to calculate specific absorbance ($SUVA_{254}$) and E4/E6. $SUVA_{254}$ is absorbance at 254 nm per unit of organic carbon, and is used as an indicator of aromaticity. High specific absorbance values indicate high aromatic organic carbon content (Hu et al., 2023). E4/E6 is the relationship between absorbance at 465 and 665 nm and is a proxy measurement for humification (Peacock et al., 2018).

Dissolved organic nitrogen (DON) was calculated by subtracting inorganic nitrogen (ammonium-N, nitrate-N and nitrite-N) concentration from total N (Yates et al., 2022). Dissolved organic phosphorous (DOP) was calculated by subtracting phosphate-P concentration from total phosphorus (TP). The proportion of total N and total P that was DON and DOP, respectively, was calculated (%DON and %DOP).

DOM: A 5 L water sample was collected in a plastic caddy and used to extract POM and DOM. A known volume of water was filtered (pre-washed VWR glass microfiber filter, 0.7μm, 47mm) to extract the particulate organic matter (POM). The filtered water was rotary evaporated (low pressure, water bath temperature 60°C) to concentrate the sample, until approximately 100mL remained. This small volume of water was placed in an evaporating dish in an oven at 60°C) until all the water had evaporated, and the residue was collected. As the water was filtered, the remaining residue was comprised of colloidal and dissolved organic matter (DOM).

DOM composition metrics

The CHNO and organic C molar concentrations from the elemental analyser were used to calculate derived metrics. With FT-ICR MS data, these metrics are calculated from the mean of all molecular formulae within a sample. For FT-ICR MS, we also calculated the total number of assigned formula and mean and standard deviation of m/z across all assigned formulae.

%C_{org}: The portion of total C that was organic C
DBE: a measure of saturation. The DBE of a compound containing only C and H will vary between 0 and 1, however DOM is more complex, containing other elements (N and O), and so DBE can only be a proxy to structure (Koch and Dittmar, 2006). DBE was negatively related to microbial respiration in water (Hu et al., 2023), and more saturated DOM in water was preferentially photo-degraded (Kujawinski et al., 2004), and had large changes when undergoing ozonation during DWT (Phungsai et al., 2018).

$$DBE = 1 + \frac{1}{2}(2(C) - (H) + (N)) \qquad \text{(Equation 1)}$$

where C, H and N are the moles of carbon, hydrogen and nitrogen in the sample. The DBE is normalized to the total number of C atoms in a molecule (DBE/C) to avoid larger molecule size affecting the result.
C_{ox}, OR, NOSC: carbon oxidation state, nominal oxidation state of carbon, and oxidative ratio (Hockaday et al., 2009; Masiello et al., 2008). These are all measures of oxidation, and can be used to infer compound class and reactivity (Boye et al., 2017; LaRowe and Van Cappellen, 2011). Highly reduced OM with low NOSC was preferentially preserved (i.e. not biodegraded) as they contained insufficient energy for microbes to use (Boye et al., 2017).

$$C_{ox} = \frac{2(O)-(H)+3(N)}{(C)} \qquad \text{(Equation 2)}$$

$$OR = 1 - \frac{C_{OX}}{4} + \frac{3(N)}{4(C)} \qquad \text{(Equation 3)}$$

$$NOSC = 4 - \left[\frac{4(C)+(H)-3(N)-2(O)}{(C)}\right] \qquad \text{(Equation 4)}$$

where C, H, N and O are the molar concentrations of carbon, hydrogen, nitrogen and oxygen in the sample
AI: aromaticity index, a measure of aromaticity. This is used to assess DOM reactivity, as highly aromatic compounds have higher rates of degradation (Hu et al., 2023; (Kim et al., 2022; Koch and Dittmar, 2006).

$$AI = \frac{(1+C-O-(0.5H))}{(C-O-N)} \qquad \text{(Equation 5)}$$

where C, H, N and O are the molar concentrations of carbon, hydrogen, nitrogen and oxygen in the sample
C/N, H/C, O/C: molar elemental ratios. These are important in soil ecological processes, and can give information about nitrogen fixation, litter decomposition and landscape processes ((He et al., 2023; Yates et al., 2019). O/C and H/C can be used to draw van Krevelen diagrams, that are used to characterise OM (Cooper et al., 2022; van Krevelen, 1950); however these can only infer 'compound-like' categories, but still give interesting information about DOM composition (Rivas-Ubach et al., 2018).
Compound class: using stoichiometric ratios of C, H, O, N and P, compounds are assigned to classes including lipids, carbohydrates, peptides, amino sugars, oxy-aromatic phytochemicals and nucleotides (using multidimensional stoichiometric compound classification method from Rivas-Ubach et al. (2018). These compound classes can be used to infer the source of DOM (e.g. algal-derived DOM has high lipid content; Shi et al. (2021)); or the susceptibility of DOM to photodegradation (e.g. high degradation rates of photo-produced aromatic compounds; Hu et al. (2023)). The nucleotide count was zero in all samples, and therefore %nucleotide was not included in further analysis.

Molecular richness: These consider the number of individual formulae in a DOM sample. Shannon-Wiener index takes molecular richness into account, whereas Gini-Simpson diversity index reflects how evenly distributed each formulae is (Tanentzap et al., 2019).

Results

Spatial Analysis

Analysis of DOM samples collected during autumn with site characteristics are shown in Table S2. For FT-ICR MS derived metrics (n=55), there were fewer land uses and vegetation covers. There were no sites with land uses of cutting or undergoing restoration, or with vegetation covers of blanket bog or improved grassland. Each analysis was carried out separately, as there were correlations between factors (e.g. land use and vegetation cover are collinear).

Land use significantly explained between 15 and 23% of variation in C/N, H/C and mean AI of DOM. DOM from sites used for peat extraction ("cutting") had higher C/N than woodland sites; whereas woodland sites had higher H/C than sites used for game reserves ("shooting"). The mean AI of DOM samples from shooting sites was also higher than woodland sites. The majority of DOM metrics had no significant differences between different land uses.

Vegetation cover significantly explained between 14 and 17% of the variation in DOM C/N, O/C, C_{ox}, OR and carbohydrate content. There were only five DOM metrics where vegetation cover was significant; the majority of DOM metrics had no significant differences between vegetation covers.

Modelling DOM metrics of samples collected during autumn showed that six parameters of physical location (latitude, longitude, elevation, catchment area, percentage peat cover and distance to sea) explained between 4 and 29% of variation Table S2). Of the 18 DOM metrics analysed, no significant models could be calculated for five metrics (%C_{org}, mean AI, Gini-Simpson, Shannon-Weiner or peptide content). Elevation was only significant in three models (H/C, O/C and carbohydrate content) and catchment area was only significant in four models (C/N, H/C NOSC and carbohydrate content). There were only four DOM metric models with adjusted R^2 higher than 0.2: carbohydrate content R^2=0.29; H/C R^2=0.24; NOSC R^2=0.21; DBE/C R^2=0.20. Each metric had a complex relationship with these parameters; most had a negative relationship with latitude and distance to the sea, and a positive relationship with longitude; supporting findings that location is important in determining DOM composition. However, these models only explained up to 29% of variation in DOM composition.

Table S1. Results of GLM analysis on group and year for all derived metrics. Significant results ($p<0.05$) are shaded grey. All metrics derived from FT-ICR MS (bottom half of table) n=55. Post-hoc Waller-Duncan k-Ratio t-tests results are shown, where groups/years with the same letter are not significantly different.

	GLM Model		Source p			Semi partial ETA R^2			Waller on Group				Waller on Year			
**Metric**	**p**	**R^2**	**Group**	**Year**	**Group*Year**	**Group**	**Year**	**Group*Year**	**1**	**2**	**3**	**4**	**2018**	**2019**	**2020**	**2021**
C/N (n=129)	0.3905	0.12	0.0148	0.5244	0.9196	0.08	0.02	0.03	A	A	AB	B	A	A	A	A
H/C (n=116)	0.0001	0.55	0.0003	0.0129	0.0001	0.09	0.05	0.24	A	B	C	BC	BC	C	AB	A
O/C (n=100)	0.0127	0.28	0.3025	0.2247	0.0584	0.01	0.01	0.07	AB	A	AB	B	A	B	AB	AB
DBE/C (n=121)	0.0001	0.38	0.0004	0.4090	0.0054	0.12	0.02	0.14	A	B	B	C	A	A	A	A
C_{ox} (n=119)	0.0237	0.21	0.2438	0.1314	0.2985	0.03	0.04	0.07	A	A	A	A	A	B	B	AB
OR (n=119)	0.0179	0.22	0.2129	0.1086	0.2978	0.03	0.05	0.07	AB	A	B	AB	B	A	A	AB
$\%C_{org}$ (n=120)	0.0001	0.35	0.0136	0.1239	0.0026	0.07	0.04	0.17	B	B	B	A	B	A	A	AB
AI (n=192)	0.0001	0.41	0.1345	0.2112	0.0001	0.04	0.04	0.30	A	A	A	A	B	A	AB	AB
mean AI	0.0153	0.39	0.5495	0.0396	0.4046	0.03	0.13	0.00	B	AB	AB	A	B	B	B	A
mean NOSC	0.0467	0.34	0.0295	0.0890	0.9793	0.15	0.11	0.01	B	B	AB	A	AB	AB	B	A
G-Simpson	0.0001	0.59	0.0297	0.0001	0.0001	0.09	0.44	0.40	A	A	A	A	B	A	A	A
Shannon	0.2456	0.25	0.2651	0.0426	0.6088	0.07	0.15	0.06	A	A	A	A	AB	AB	B	A
lipid %	0.0305	0.36	0.0436	0.0871	0.8177	0.13	0.10	0.03	A	A	AB	B	AB	A	A	B
carb %	0.0010	0.49	0.0003	0.0452	0.6791	0.28	0.10	0.04	C	BC	A	AB	AB	B	AB	A
amino %	0.0016	0.47	0.1031	0.0003	0.6634	0.08	0.28	0.04	A	A	A	A	A	A	A	B
peptide %	0.0027	0.45	0.0297	0.0104	0.7818	0.13	0.16	0.03	C	BC	AB	A	B	B	B	A
oxy-aro. %	0.1418	0.29	0.1001	0.3425	0.7120	0.11	0.06	0.05	AB	B	AB	A	A	A	A	A
DBE/C	0.0072	0.42	0.0122	0.0208	0.9733	0.17	0.15	0.01	C	BC	AB	A	B	B	B	A

Table S2. Results of GLM analysis on land use or vegetation cover for all derived metrics, and regression using latitude, longitude, elevation, catchment area, percentage peat cover and distance to sea. Significant results ($p<0.05$) are shaded grey. All metrics derived from FT-ICR MS (bottom half of table) n=55. These analyses included all samples collected in autumn (2018-2021).

	**GLM: Land use[1]**				**GLM: Veg cover[2]**				**Regression[3]**								
**Metric**	**p**	**R^2**	**high**	**low**	**P**	**R^2**	**high**	**low**	**p**	**adj. R^2**	**intercept**	**Lat.**	**Long.**	**Ele.**	**Cat.**	**Peat.**	**Dist. Sea**
C/N (n=129)	0.0001	0.23	C	W	0.0012	0.17	BB	AG	0.0001	0.1704	282.51	-4.27	2.97	-	-0.35	0.27	-0.59
H/C (n=116)	0.0072	0.16	W	S	0.0556	0.10	IG	AG	0.0001	0.24	2.32	-	0.07	-0.003	0.008	-	0.005
O/C (n=100)	0.0567	0.14	W	F	0.0117	0.16	IG	H	0.0055	0.08	2.19	-	-	-0.0005	-	-0.01	-
DBE/C (n=121)	0.1710	0.09	R	S	0.2800	0.06	BB	HG	0.0001	0.17	-2.7	0.07	-	-	-	-0.004	0.003
C_{ox} (n=119)	0.2463	0.08	W	S	0.0035	0.16	CW	H	0.0296	0.04	2.9	-	0.06	-	-	-0.02	-
OR (n=119)	0.3132	0.07	S	W	0.0038	0.16	H	CW	0.0291	0.04	0.32	-	-0.01	-	-	0.004	-
%C_{org} (n=120)	0.1537	0.09	S	F	0.1468	0.08	BW	AG	0.2284	0.01							
AI (n=92)	0.3584	0.09	S	R	0.4084	0.07	BW	IG	0.0004	0.17	34.31	-0.56	0.26	-	-	0.009	-0.05
mean AI	0.0440	0.20	S	W	0.7247	0.04	AG	BW	0.0880	0.05							
mean NOSC	0.2341	0.13	S	M	0.1336	0.13	H	BW	0.0046	0.21	6.73	0.13	0.07	-	-0.002	0.005	-0.009
G-Simpson	0.7962	0.05	M	F	0.6036	0.05	H	AG	0.1753	0.05							
Shannon	0.9214	0.03	S	F	0.5106	0.06	H	BW	0.1541	0.04							
lipid %	0.2395	0.13	W	S	0.2209	0.11	BW	H	0.0129	0.16	-352.06	7.06	-3.75	-	-	-0.37	0.51
carb %	0.1277	0.16	F	W	0.0449	0.17	AG	BW	0.0005	0.29	4.42	-0.06	-	0.001	0.004	-0.005	-0.005
amino %	0.7319	0.05	S	W	0.5230	0.06	HG	BW	0.1269	0.03							
peptide %	0.9261	0.03	S	P	0.5424	0.06	CW	BW	0.0034	0.13	11.95	-0.2	-	-	-	-	-
oxy-aro. %	0.1599	0.15	S	W	0.1795	0.12	H	BW	0.0109	0.16	355.81	-5.54	2.93	-	-	0.38	-0.42
DBE/C	0.0741	0.18	S	W	0.2722	0.10	H	BW	0.0039	0.20	2.43	-0.04	0.02	-	-	0.002	-0.002

1. Land use abbreviations: C = cutting, F = felled forest, G = grazing, M = moorland, P = plantation, R = restored, S = shooting, W = woodland

2. Vegetation cover abbreviations: AG = acid grassland, BB = blanket bog, BW = broadleaf woodland, CW = coniferous woodland, H = heather, HG = heather grassland, IG = improved grassland

3. Regression values shown are parameter estimates. Parameter abbreviations: Lat. = latitude, Long. = longitude, Ele. = elevation (m asl), Cat. = catchment area (km^2), Peat. = percentage peat cover, Dist. Sea = distance to sea (km)

Temporal Analysis

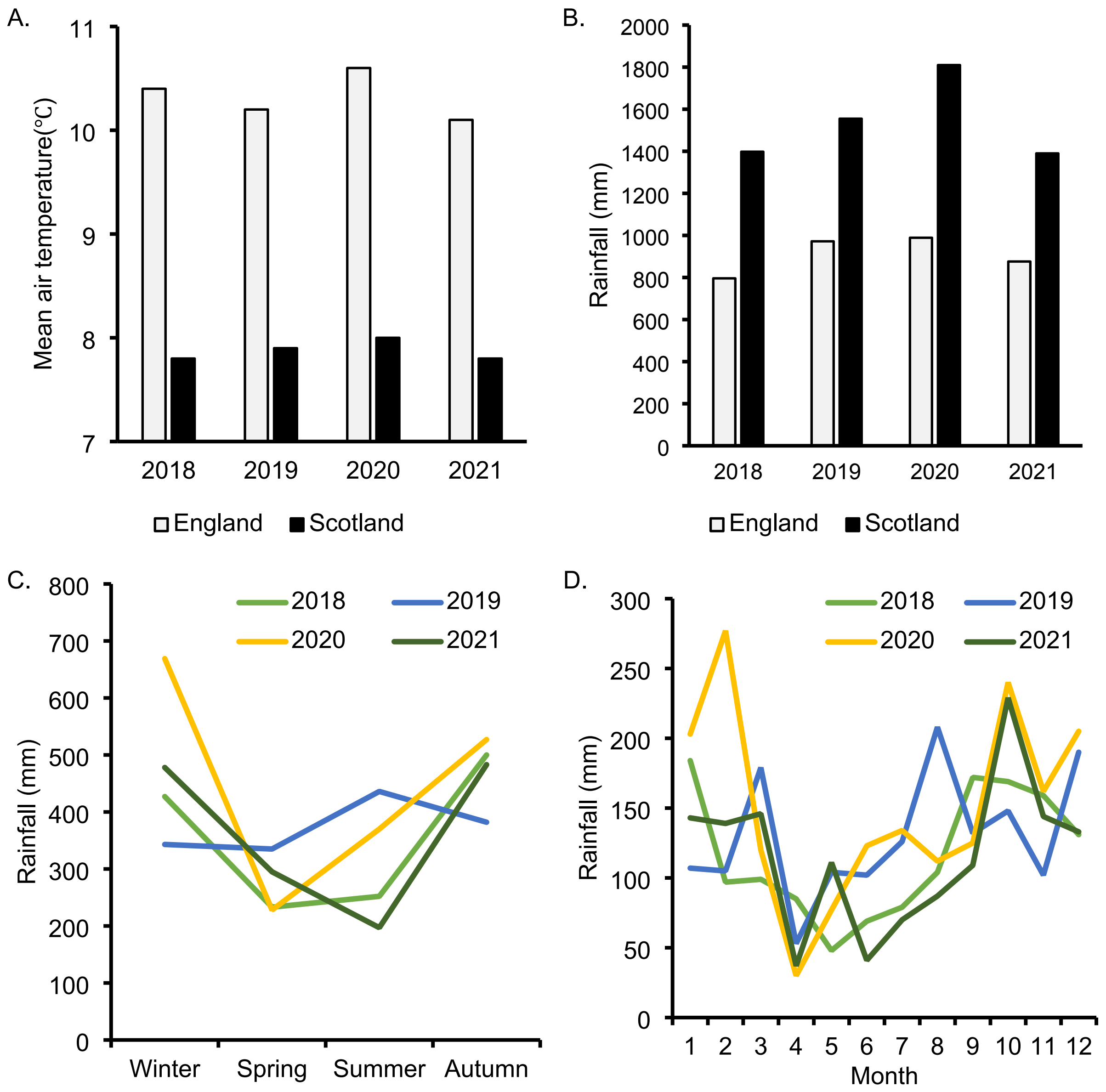

Figure S1. UK Met Office data for 2018-2021 (Kendon et al., 2019; Kendon et al., 2020; 2021; Kendon et al., 2022), for England and Scotland: A) Average air temperatures, and B) total rainfall. Monthly rainfall (mm) in (C) each season, and (D) calendar month (1=Jan, 12=Dec), from 2018-2021 in Scotland.

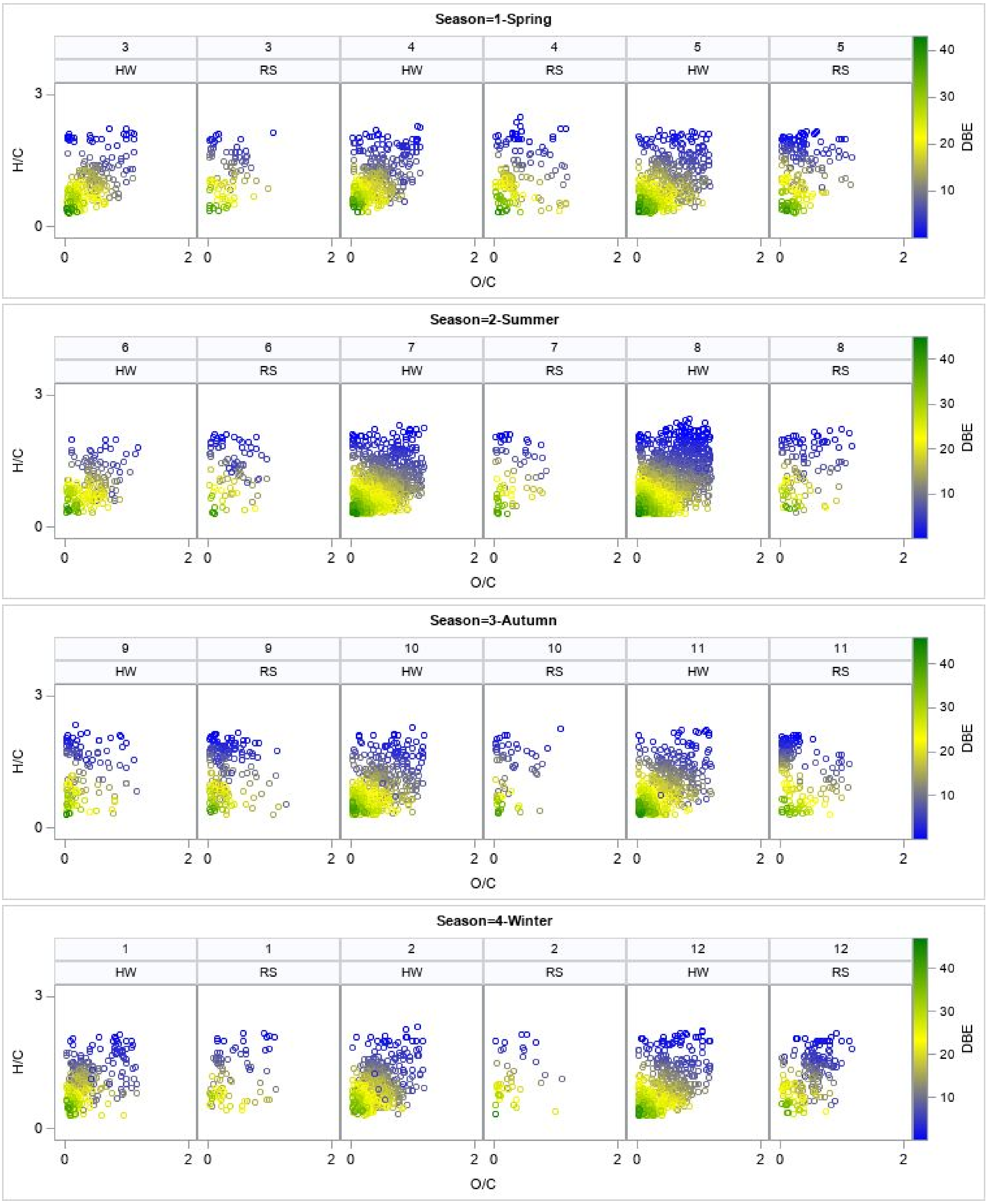

Figure S2. Van Krevelen plots (O/C against H/C) of unique compounds in Group 4 reservoir (RS) and headwater (HW) DOM samples, by month (numbered 1-12) in each season (Spring, Summer, Autumn, Winter). In each month, there were a higher number of unique compounds found in HW DOM than in RS DOM. Each point represents a single compound found only in that DOM sample, and is coloured to reflect its double bond equivalent (DBE) from blue (low DBE) to green (high DBE).

Analysis of the molecular formulae found in reservoirs can be targeted at specific compounds, such as geosmin ($C_{12}H_{22}O$) and 2-methylisoborneol ($C_{11}H_{20}O$, '2-MIB'); both are known to cause taste and odour issues in drinking water supplies (Shi et al., 2021). Neither compound was found in DOM from the reservoir sampled for this study on any sampling occasion. Compounds with similar molecular formulae were found (e.g. $C_{12}H_{16}O$, $C_{12}H_{22}O_2$, $C_{11}H_{20}O_2$), showing that compounds with low C and O content were detected by FT-ICR MS, but not geosmin or 2-MIB specifically.
Using FT-ICR MS to look for key molecular formulae (such as DBP-precursors) was investigated by Herzsprung et al. (2023), who used at a semi-quantitative approach to find relative intensity differences of key molecular formulae over time or space. They identify a photo-product $C_{10}H_{14}O_6$, a precursor to DBPs, and a poly-phenol $C_{19}H_{14}O_{13}$, likely to be photo-degraded, and track their intensity in a reservoir over depth and time. Both these compounds were found in the reservoir and headwater in this study on every sampling occasion (12 times). $C_{10}H_{14}O_6$ can be chlorinated to $C_{10}H_{12}O_6Cl_2$ by electrophilic substitution during DWT (Phungsai et al., 2016). $C_{19}H_{14}O_{13}$ was found in drinking water supplies in Sweden and decreased after $Al_2(SO_4)_3$ flocculation treatment, and both increased and decreased after NH_2Cl/UV disinfection at two different DWT plants (Lavonen et al., 2015). Compounds associated with taste and odour issues (geosmin and 2MIB) were not found in reservoir OM in this study, however, compounds known to be precursors to DBPs were identified in both headwater and reservoir OM. Using FT-ICR MS to find specific compounds present in raw water supplies can therefore be useful to water companies.

The combination of climate and weather factors that lead to different DOM compositions also impacted on water chemistry e.g. the average Mn concentration was more than twice as high in 2021 than 2018-2020 (0.039 mg l^{-1} compared to 0.013-0.017 mg l^{-1}). The number of unique compounds in DOM in reservoirs was higher when heavy metals including Mn were high (Table 2). Increasing Mn concentrations are having wider implications for the water industry and DWT (Graham et al., 2012; Semasinghe and Rousso, 2023), and could also indicate a higher number of unique compounds in DOM, potentially leading to an increase in more-difficult to treat DOM. As climate changes, the likelihood of warm summers increases, leading to high Mn concentrations, and more diverse DOM in drinking water supplies, alongside lower water volumes available to abstract, increasing demands on the water industry.

References

Boye, K., Noël, V., Tfaily, M.M., Bone, S.E., Williams, K.H., Bargar, J.R. and Fendorf, S. 2017. Thermodynamically controlled preservation of organic carbon in floodplains. Nature Geoscience 10(6), 415-419.

Cooper, W.T., Chanton, J.C., D'Andrilli, J., Hodgkins, S.B., Podgorski, D.C., Stenson, A.C., Tfaily, M.M. and Wilson, R.M. 2022. A history of molecular level analysis of natural organic matter by FTICR mass spectrometry and the paradigm shift in organic geochemistry. Mass Spectrometry Reviews 41(2), 215-239.

Graham, M.C., Gavin, K.G., Kirika, A. and Farmer, J.G. 2012. Processes controlling manganese distributions and associations in organic-rich freshwater aquatic systems: the example of Loch Bradan, Scotland. Science of the total environment 424, 239-250.

He, C., Yi, Y., He, D., Cai, R., Chen, C. and Shi, Q. 2023. Molecular composition of dissolved organic matter across diverse ecosystems: Preliminary implications for biogeochemical cycling. Journal of Environmental Management 344, 118559.

Herzsprung, P., Kamjunke, N., Wilske, C., Friese, K., Boehrer, B., Rinke, K., Lechtenfeld, O.J. and von Tümpling, W. 2023. Data evaluation strategy for identification of key molecular formulas in dissolved organic matter as proxies for biogeochemical reactivity based on abundance differences from ultrahigh resolution mass spectrometry. Water Research 232, 119672.

Hockaday, W., Masiello, C., Randerson, J., Smernik, R., Baldock, J., Chadwick, O. and Harden, J. 2009. Measurement of soil carbon oxidation state and oxidative ratio by ^{13}C nuclear magnetic resonance.

Hu, J., Kang, L., Li, Z., Feng, X., Liang, C., Wu, Z., Zhou, W., Liu, X., Yang, Y. and Chen, L. 2023. Photo-produced aromatic compounds stimulate microbial degradation of dissolved organic carbon in thermokarst lakes. Nature Communications 14(1), 3681.

Kendon, M., McCarthy, M., Jevrejeva, S., Matthews, A. and Legg, T. 2019. State of the UK climate 2018. International Journal of Climatology 39, 1-55.

Kendon, M., McCarthy, M., Jevrejeva, S., Matthews, A., Sparks, T. and Garforth, J. 2020. State of the UK Climate 2019. International Journal of Climatology 40, 1-69.
Kendon, M., McCarthy, M., Jevrejeva, S., Matthews, A., Sparks, T. and Garforth, J. 2021. State of the UK Climate 2020. International Journal of Climatology 41, 1-76.
Kendon, M., McCarthy, M., Jevrejeva, S., Matthews, A., Sparks, T., Garforth, J. and Kennedy, J. 2022. State of the UK Climate 2021. International Journal of Climatology 42, 1-80.
Kim, S., Kim, D., Jung, M.J. and Kim, S. 2022. Analysis of environmental organic matters by Ultrahigh-Resolution mass spectrometry—A review on the development of analytical methods. Mass spectrometry reviews 41(2), 352-369.
Koch, B.P. and Dittmar, T. 2006. From mass to structure: An aromaticity index for high-resolution mass data of natural organic matter. Rapid communications in mass spectrometry 20(5), 926-932.
Kujawinski, E.B., Del Vecchio, R., Blough, N.V., Klein, G.C. and Marshall, A.G. 2004. Probing molecular-level transformations of dissolved organic matter: insights on photochemical degradation and protozoan modification of DOM from electrospray ionization Fourier transform ion cyclotron resonance mass spectrometry. Marine Chemistry 92(1-4), 23-37.
LaRowe, D.E. and Van Cappellen, P. 2011. Degradation of natural organic matter: a thermodynamic analysis. Geochimica et Cosmochimica Acta 75(8), 2030-2042.
Lavonen, E., Kothawala, D., Tranvik, L., Gonsior, M., Schmitt-Kopplin, P. and Köhler, S. 2015. Ultra-high resolution mass spectrometry explains changes in the optical properties of dissolved organic matter during drinking water production. Water Research 85, 286-294.
Masiello, C., Gallagher, M., Randerson, J., Deco, R. and Chadwick, O. 2008. Evaluating two experimental approaches for measuring ecosystem carbon oxidation state and oxidative ratio. Journal of Geophysical Research: Biogeosciences 113(G3).
Peacock, M., Jones, T.G., Futter, M.N., Freeman, C., Gough, R., Baird, A.J., Green, S.M., Chapman, P.J., Holden, J. and Evans, C.D. 2018. Peatland ditch blocking has no effect on dissolved organic matter (DOM) quality. Hydrological Processes 32(26), 3891-3906.
Phungsai, P., Kurisu, F., Kasuga, I. and Furumai, H. 2016. Molecular characterization of low molecular weight dissolved organic matter in water reclamation processes using Orbitrap mass spectrometry. Water research 100, 526-536.
Phungsai, P., Kurisu, F., Kasuga, I. and Furumai, H. 2018. Changes in dissolved organic matter composition and disinfection byproduct precursors in advanced drinking water treatment processes. Environmental science & technology 52(6), 3392-3401.
Rivas-Ubach, A., Liu, Y., Bianchi, T.S., Tolić, N., Jansson, C. and Paša-Tolić, L. 2018. Moving beyond the van Krevelen Diagram: A New Stoichiometric Approach for Compound Classification in Organisms. Analytical Chemistry 90(10), 6152-6160.
Semasinghe, C. and Rousso, B.Z. 2023. In-Lake Mechanisms for Manganese Control—A Systematic Literature Review. Sustainability 15(11), 8785.
Shi, W., Zhuang, W.-E., Hur, J. and Yang, L. 2021. Monitoring dissolved organic matter in wastewater and drinking water treatments using spectroscopic analysis and ultra-high resolution mass spectrometry. Water Research 188, 116406.
Tanentzap, A.J., Fitch, A., Orland, C., Emilson, E.J.S., Yakimovich, K.M., Osterholz, H. and Dittmar, T. 2019. Chemical and microbial diversity covary in fresh water to influence ecosystem functioning. Proc Natl Acad Sci U S A 116(49), 24689-24695.
van Krevelen, D. 1950. Graphical-statistical method for the study of structure and reaction processes of coal. Fuel 29, 269-284.
Yates, C.A., Johnes, P.J., Brailsford, F.L., Evans, C.D., Evershed, R.P., Glanville, H.C., Jones, D.L., Lloyd, C.E., Marshall, M.R. and Owen, A.T. 2022. Determining patterns in the composition of dissolved organic matter in fresh waters according to land use and management. Biogeochemistry, 1-20.
Yates, C.A., Johnes, P.J., Owen, A.T., Brailsford, F.L., Glanville, H.C., Evans, C.D., Marshall, M.R., Jones, D.L., Lloyd, C.E. and Jickells, T. 2019. Variation in dissolved organic matter (DOM) stoichiometry in UK freshwaters: assessing the influence of land cover and soil C: N ratio on DOM composition. Limnology and Oceanography 64(6), 2328-2340.
